# Exploring the County Level Mortality Pattern Variations in Rural Areas of Iran (2006-2016)

**DOI:** 10.34172/ijhpm.2024.8032

**Published:** 2024-06-12

**Authors:** Mehrangiz Rezaee, Nader Tavakoli, Siamak Tahmasbi

**Affiliations:** ^1^Department of Geography, Yazd University, Yazd, Iran.; ^2^Trauma and Injury Research Centre, Iran University of Medical Sciences, Tehran, Iran.; ^3^Centre for Remote Sensing & GIS Research, Faculty of Earth Sciences, Shahid Beheshti University, Tehran, Iran.

**Keywords:** Mortality Rate, Spatial Autocorrelation, Spatial Clustering, Rural Areas, Iran

## Abstract

**Background:** Mortality rate in rural areas is a useful measure of the health of the population and the function of the health system, which varies over space and time. The objective of this research is to explore the spatial and temporal variations in the rural mortality rate in Iran at the county level in 2006, 2011, and 2016.

**Methods:** data were gathered from the rural population and mortality statistics published by the Statistical Centre of Iran (SCI) and the National Organization for Civil Registration (NOCR). Global spatial patterns were assessed using the global Moran’s I and local clusters through the local Moran’s I.

**Results:** Spatial distribution of rural mortality rate shows that during the years under study the number of counties with a lower rate has increased. The counties with rate of less form continuous areas in the southwest, central and east regions. The excess risk map reveals significant variations in both value and extent. Also, the values of Moran’s index increased from 0.1848 in 2006 to 0.4041 in 2016, which indicates the strengthening of the cluster spatial pattern of the overall rural mortality rate. Local patterns have undergone substantial changes over space and time.

**Conclusion:** The findings indicate significant spatial and temporal variations in rural mortality rates in Iran. Policy-makers can use this information to plan and enhance healthcare infrastructure in specific counties. The findings serve for evaluating the effectiveness of health policies, enabling policy-makers to make informed decisions, allocate resources efficiently and design targeted interventions for improved public health outcomes.

## Background

**Key Messages**

**Implications for policy makers**

- Analyzing mortality spatially and temporally offers insights into the variations of mortality in rural areas, providing policy-makers with a valuable perspective. This information aids in the assessment of previous policies and informs the design of future strategies.
- Designing targeted interventions for prevention and providing healthcare in rural areas with high mortality rates.
- Conducting evidence-based studies on economic-social and environmental factors that determine mortality in rural areas.

**Implications for the public**

This study suggests that spatial patterns of mortality rates in rural areas have changed spatially and temporally. the results of the spatial autocorrelation analysis obtained from the global Moran’s I index are positive for all three years, which shows positive spatial autocorrelation. Also, the values of Moran’s index indicate the strengthening of the cluster spatial pattern of rural mortality rate. The findings indicate that mortality rates in rural areas exhibit dynamic spatial patterns that vary over time. Adopting a geographic approach in exploring spatial heterogeneity and mortality patterns is an inevitable necessity in allocate resources efficiently, determining public health priorities and interventions.

Mortality rate is a key indicator of overall health in the country.^1^ People residing in rural regions frequently encounter increased public health difficulties due to limited access to healthcare services, lower likelihood of having health insurance, and a higher probability of experiencing poverty.^2^ The enduring disparity between rural and urban areas and the prevalence of severe rural poverty have resulted in the poor health condition of rural populations. Rural regions continued to exhibit significant poverty and underdevelopment, leading to notably poor health measures.^3^ Therefore, exploring the spatial patterns of rural mortality rates plays an important role in the formulation of valid a etiological hypotheses that help us to better understand the relationships between events of interest and factors contributing to their occurrence.^4^ Moreover, the mapping of the geographical variation in risk may help to identify areas with lower or higher rates of incidence with respect to a reference average. Estimating the regional mortality rate is used to making disease maps, investigate the relationship between the disease rate and social, demographic and environmental factors.^5,6^ This can help us in allocating healthcare resources, taxes, public services, designing life insurance and pension plans.^5,7^

 Mortality data are frequently presented at the overall population level, possibly obscuring small-scale variations over time and space and between different population sub-groups.^8^ In the two last decades, however, improvements in social and health registries, developments of computing technology and the availability of geographical information systems have made it possible to use smaller areas with much finer geographical resolution. Studies at the small-area level have provided a more precise approach to describe spatial patterns of causes of death, to investigate spatially the relationship between social and environmental determinants and health, and to establish health priorities.^9^

 In particular, small-area mortality atlases published in several countries have not only showed general patterns in the mortality data but have also identified specific high-risk areas.^10^ Three good recent examples are the Atlas of United States Mortality, which was the first publication of maps of all leading causes of death in the United States on a small-area scale, death in Britain, which shows how local mortality rates have changed between 1950 and 1990 in Britain at the small-area level, and The Spanish Atlas of Mortality includes multiple maps for the most important causes of death by gender and age as well as graphs with data by Autonomous Community.^9,11,12^

 Examining the historical patterns of global mortality rates throughout the past two centuries reveals a significant decrease worldwide.^13^ Similarly, mortality rates decreased dramatically over the period in Iran,^14^ with its crude mortality rate dropping from 32.8 in 1921 to 5.8 in 2006, further reducing to 5.17 in 2011 and 4.63 in 2016.^15^ Studies indicated that there were variations in both the rates and declines across different provinces.^15-17^ In Iran, no study has so far systematically examined the spatial patterns of mortality in rural areas. Spatial and temporal patterns of overall mortality rates in rural areas remain unknown, and there are limited and case studies in this area. In this study, using spatial statistics analysis, we have focused on the study of rural mortality in the counties of Iran, which are different in terms of social, cultural, geographical, and economic factors. Because of these differences, it was considered that rural mortality could create different clusters. Most limited studies on rural mortality in Iran or abroad have been conducted using traditional statistical techniques, without considering the spatial dimension. Also, aggregating demographic data at the macro level ignores local patterns, which can be misleading. However, understanding the spatial pattern of rural mortality helps to assess local effects on mortality by geographical analysis, hypothesizing and identifying the causes of mortality, and taking immediate action. Based on these motivations, this article seeks to explore patterns and study the spatial and temporal variations of mortality in rural areas of Iran (2006-2016).

## Methods

**Figure 1:**
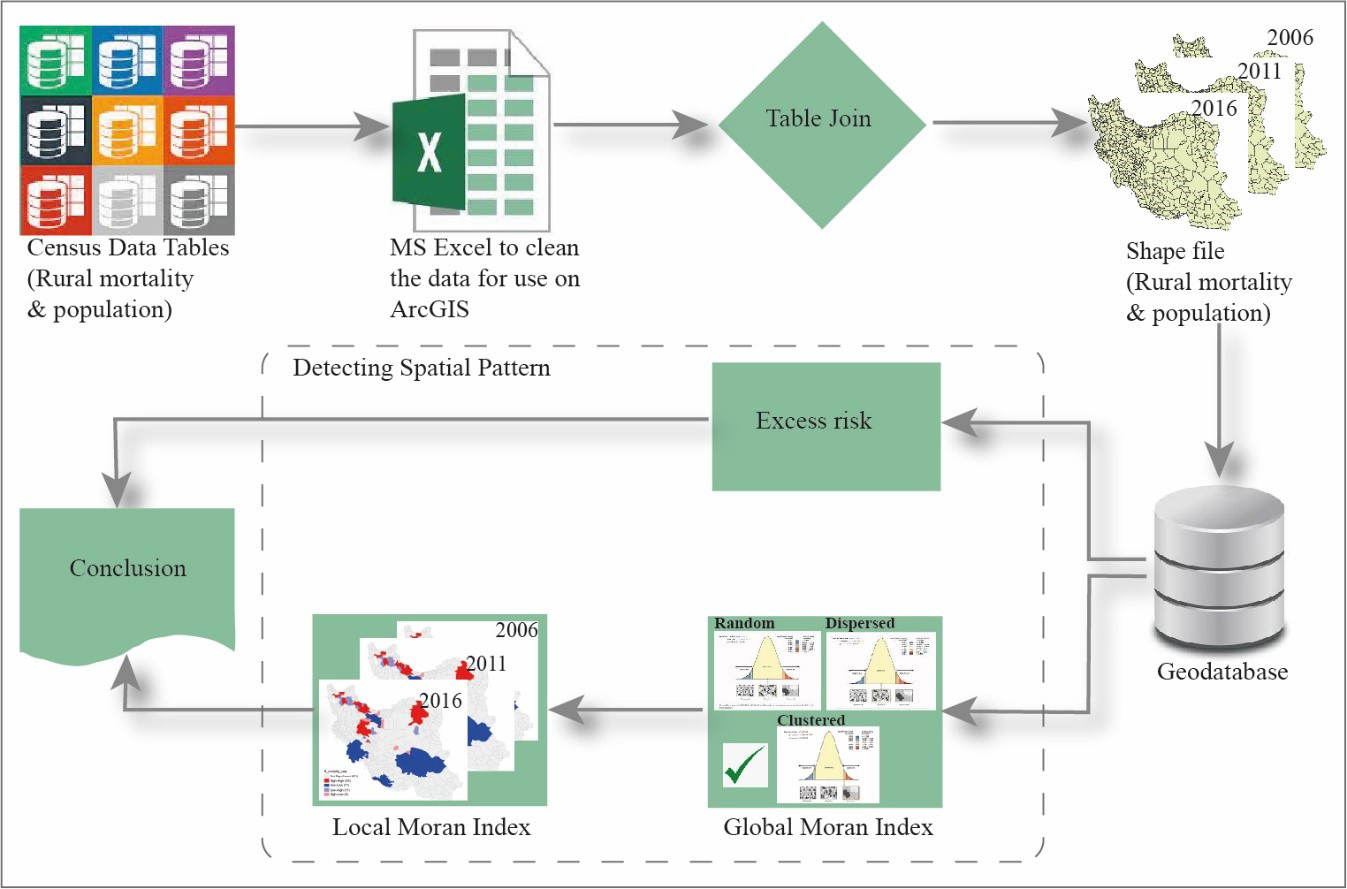
Flowchart for Detecting Spatial Patterns of Rural Mortality.

 The selection of a spatial unit for data collection and analysis plays an important role in the spatial analysis process.^18^ In this study, we use data for 428 Iran counties that is based on the latest census of 2016. The process for detecting spatial patterns often involves several key steps that presented in Figure 1.

1. Data collection: One of the main limitations in developing countries is the lack of access to high-quality data, which makes spatio-temporal analysis challenging. Considering that population and housing censuses has conducted in 2006, 2011, and 2016, also access to spatial data from 2006 onwards, the data relevant to counties used in this study is compiled from the rural population and mortality statistics published by the Statistical Centre of Iran (SCI) and National Organization for Civil Registration (NOCR) for the years 2006, 2011, and 2016.
2. Data preprocessing: The data were cleaned to handle any errors, inconsistencies, and missing data in the Excel, which includes removing outliers, standardizing the data format. Finally, the data were prepared to join the shapefile of the counties, then they were joined to the shapefile based on the unique ID code in the ArcGIS environment and a geodatabase was created.
3. Detecting spatial pattern: There are different spatial and non-spatial techniques to reveal patterns in the dataset, for this purpose both spatial and non-spatial methods were used. Data visualization is one of the most common methods, for this purpose, the spatial distribution of rural mortality was mapped in the ArcGIS environment. Also, the excess risk was used as a non-spatial method. Due to the limitations of non-spatial methods, spatial methods such as global Moran’s I and local Moran’s I for extracting were used.

 The index investigated in this study is the rural mortality rate, Mortality rate is typically expressed in units of deaths per 1000 individuals per year.^19^ In addition to the mortality rate, a commonly used notion in demography and public health analysis is the concept of a standardized mortality rate, sometimes also referred to as relative risk or excess risk.^20-22^ The idea is to compare the observed mortality rate to a national (or regional) standard. More specifically, the observed number of events is compared to the number of events that would be expected had a reference risk been applied.^20,21^ Formally, this is expressed as^23-27^:


(1) $$\pi =\frac{\sum_{i=1}^{i=n}O_{i}}{\sum_{i=1}^{i=n}P_{i}}$$

Where *O*_i_ is observed events and *P*_i_ is populations in region i=1, …, n, which yields the expected number of mortalities for each area i as:


(2) $$E_{i}=\pi *P_{i}$$

The relative risk then follows as the ratio of the observed number of mortalities:


(3) $$SMR_{i}=\frac{O_{i}}{E_{i}}$$

In spatial data analysis, it is often necessary to determine whether there are identifiable spatial patterns. There are several ways to test the existence of such patterns.^28^ Generally, two methods of global and local spatial autocorrelation are used to measure the spatial pattern.

 Spatial autocorrelation is a general geographical phenomenon in nature, which indicates spatial association and spatial dependence of geographic object.

###  Global Spatial Autocorrelation

 Global spatial autocorrelation measures the degree of dependence between observations in geographic space.^29^ Moran’s I statistic is often used for this purpose. This index measures the tendency of events to cluster by identifying the points having similar attribute values and spatially close to each other.^30,31^ It is also used to test the null hypothesis, the null hypothesis for Moran’s I is that there is no spatial autocorrelation,^28,32^ or the spatial pattern of the data is random^33^ If the null hypothesis is rejected, there is spatial autocorrelation.^30^ Often social science variables tend to be spatially positively autocorrelated due to the way phenomena are organized geographically. Demographic and socio-economic characteristics are good examples of variables that show positive spatial autocorrelation.^34^ The calculation formula of Moran’s I is given below.


(4) $$I=\frac{\sum_{i=1}^{n}\sum_{j=1}^{n}w_{ij}\left(x_{i}-\bar{X}\right)\left(x_{j}-\bar{X}\right)}{\left(\sum_{i=1}^{n}\sum_{j=1}^{n}w_{ij}\right)\;\sum_{i=1}^{n}{\left(x_{i}-\bar{X}\right)}^{2}}$$

The observed values associated with positions i and j are denoted by the rural mortality rates in spatial units (counties) *x*_i_and *x*_j_, respectively. $\bar{X}$ represents the average rural mortality rate in the study area. The weight matrix is represented by *w*_ij_, and the number of samples (counties) is represented by *n*.^35^

 The Moran’s I index will be a value between -1 and 1. Positive spatial autocorrelation will show values that are clustered. Negative autocorrelation is dispersed. Random is close to zero.

###  Local Spatial Autocorrelation

 Local spatial autocorrelation is a local clustering effect due to the similarity of observed values in a contiguous neighborhood. To describe the spatial heterogeneity of spatial autocorrelation, we must rely on measures that reveal spatial autocorrelation at the local scale.^36^ Local spatial autocorrelation indicates spatial clustering and corresponds to non-stationarity, which implies that underlying socio-economic context processes (such as wealth, occupation, gender, ethnicity and social class, which in turn correlates with factors such as environmental exposures, educational quality, and access to healthcare) cause spatial heterogeneity in the study are.^37^ Global Moran’s I statistic cannot reveal the details of spatial clusters in spatial autocorrelation.^29,38,39^ The local index of spatial dependence allows researchers to examine local variations in spatial dependence, and to answer the question of where spatial autocorrelation exists.^29^ Local Moran’s I is often used to measure local spatial autocorrelation.^40^ The calculation formula of Moran’s I is given below.^41^


(5) $$I_{i}=\frac{x_{i}-\mu }{\sum_{i}^{n}{\left(x_{i}-\mu \right)}^{2}}\;\sum_{j}^{n}W_{ij\left(x_{j}-\mu \right)}$$

The map of local Moran’s statistic also known as Local Indicators of Spatial Association (LISA) map or cluster and outlier analysis. LISA map shows 5 patterns based on each place and its neighborhood unit. A positive value for I indicates that a feature has neighboring features with similarly high or low attribute values; this feature is part of a cluster Spatial clusters include high-high clusters (high values is surrounded primarily by high values) and low-low clusters (low values is surrounded primarily by low values). A negative value for I indicates that a feature has neighboring features with dissimilar values; this feature is an outlier that include high-low clusters (high values is surrounded primarily by low values) and low-high clusters (low values is surrounded primarily by high values).^38,42^ For both global and local statistics, the spatial weight must be defined in order to add spatiality to the models. Because the matrix has the ability to affect the outcomes, how it is designed is crucial.^43^ Different approaches are used to define the neighborhood and build the spatial weight matrix. However, choosing the type of spatial weight matrix is one of the most difficult and controversial methodological issues in spatial data exploratory analysis.^44^ The nearest neighbor (k-NN) method was used to create the weight, and to select the optimal number of neighbors, the number of different neighborhoods was tested, and among them, the number of 8 neighborhoods that had the highest z-score was chosen as the optimal number.

## Results

**Figure 2:**
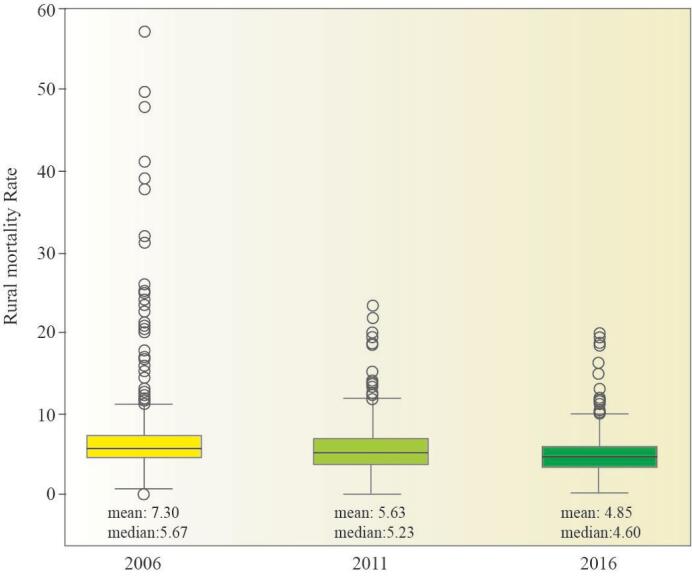
Boxplot of Rural Mortality Rate of Iran (2006, 2011, and 2016).

 In Figure 2, which is related to box plot of rural mortality rate for the three years of 2006, 2011, and 2016, it can be seen that the distribution for all three years has an almost symmetrical normal distribution and data for 2016 has less dispersion (standard devotion) compared to the other two years. In addition, the median value (second quartile) is also lower this year compared to the other two years. According to the available information, the average rural mortality rate was 7.30 in 2006, which decreased to 5.63 in 2011 and 4.85 in 2016 (Figure 2).

**Figure 3:**
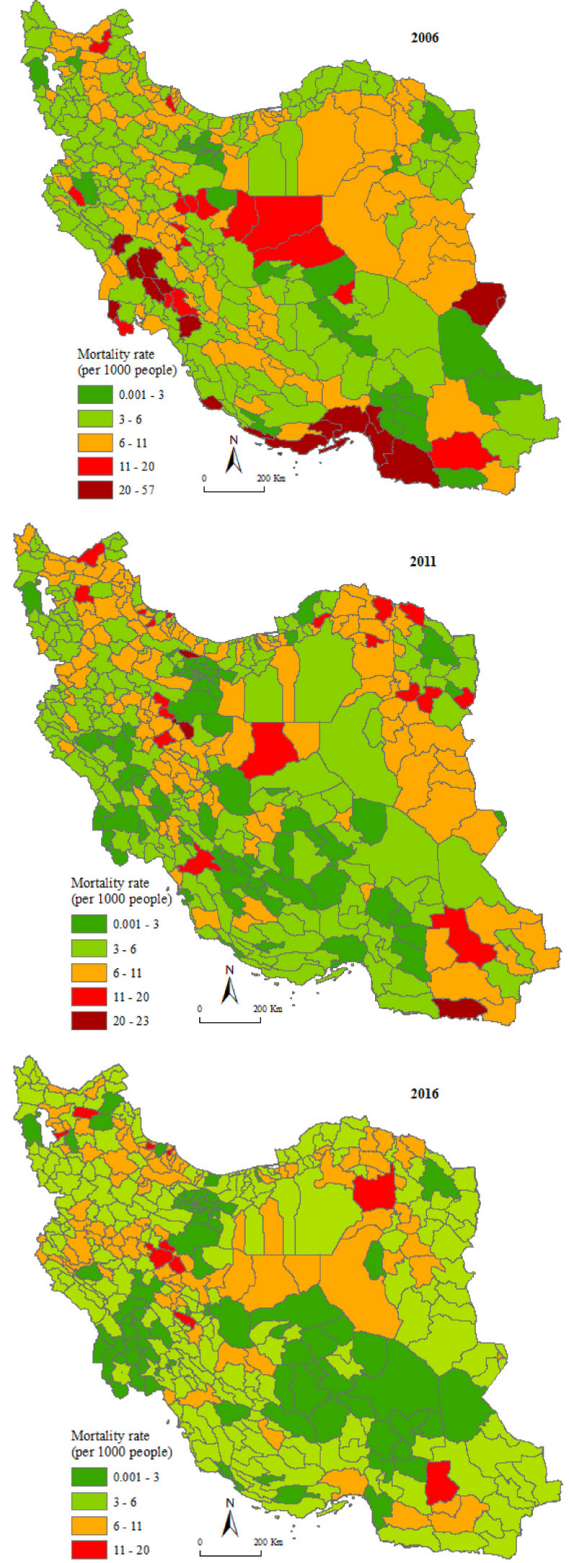
Geographical Distribution Rural Mortality Rate of Iran (2006, 2011, and 2016).

 The rural mortality rate at the county level for the three years of 2006, 2011, and 2016 is shown in Figure 3. In 2006, the southern and southwestern regions of Iran have the highest rural mortality rates. A number of central counties are also in the upper classes of rural mortality rates. In 2011, the mortality situation in these areas has decreased and placed in the low rate classes. Geographical distribution of rural mortality rate indicates that during the years under study the number of counties with a lower mortality rate has increased.

 The map of rural mortality in 2016 shows that the counties with a rural mortality rate of less than 3 form continuous and wide areas in the southwest, central and Southeast regions. Also, the number of counties that are in the class with high mortality rate has decreased and it is observed that there is no county with a rural mortality rate higher than 20 in 2016.

###  Excess Risk of Rural Mortality

 Excess risk is defined as a ratio of the observed number of rural mortalities over the expected number of rural mortalities for a county. The expected number of rural mortalities is a number indicating what the number of deaths in the county would have been if that county had the same death rate as the country. Therefore, the country has an excess rate of 1. Moreover, values of the excess risk less than 1 indicate the counties with fewer than expected mortalities; values greater than 1 indicate the counties where the number of mortalities exceeds the expectation.^16,20^

**Figure 4:**
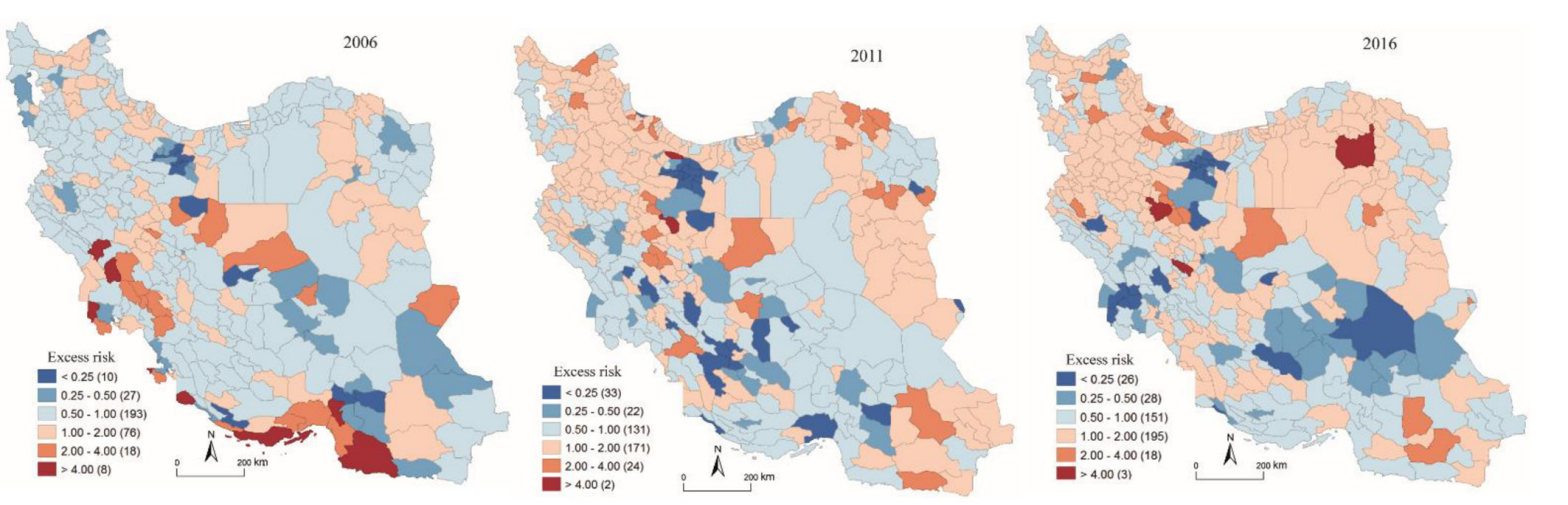
Excess Risk Maps of Rural Mortality of Iran (2006, 2011, and 2016).

 The maps of the excess risk of rural mortality show major changes both in terms of value and spatial between 2006 and 2016 (Figure 4). The number of counties whose excess risk is higher than the country excess rate is 102, 197, and 216 counties in 2006, 2011, and 2016. In 2006, the southern, southwestern and central counties have higher values than the national average. In 2011, most of these counties have values lower than the national average, and most of the northwestern, central, eastern, and southeastern counties are placed in classes with values higher than one. This trend continues in 2016, and the only noteworthy point is that in this year, the northern cities of Kerman and Sistan & Baluchistan provinces are placed in the categories of less than 0.5.

 Excess risk is a non-spatial method that ignores the effect of spatial autocorrelation. The global Moran’s index investigates the spatial autocorrelation of the rural mortality rate among the counties.

###  Global Spatial Autocorrelation of Rural Mortality Rate (2006, 2011, and 2016)

 The results of Moran’s global spatial autocorrelation are positive and significant at *P*< .001 for all three years, which shows positive spatial autocorrelation. Therefore, the null hypothesis of spatial independence of the rural mortality rate is rejected. As a result, there is a spatial dependence in the data on mortality rates, which shows clusters of high or low rural mortality rates. The positive value of Moran’s I enables us to infer those counties exhibiting high mortality rates are in proximity to counties sharing similar characteristics. Similarly, counties with low rates are situated near other counties displaying the same pattern. Also, the values of Moran’s index increased from 0.1848 in 2006 to 0.4041 in 2016, which indicates the strengthening of the cluster spatial pattern of the overall rural mortality rate. Because whenever the Moran coefficient tends to 1, it indicates that the data spatial pattern is clustered.

###  Local Spatial Autocorrelation of Rural Mortality Rate (2006, 2011, and 2016)

**Figure 5:**
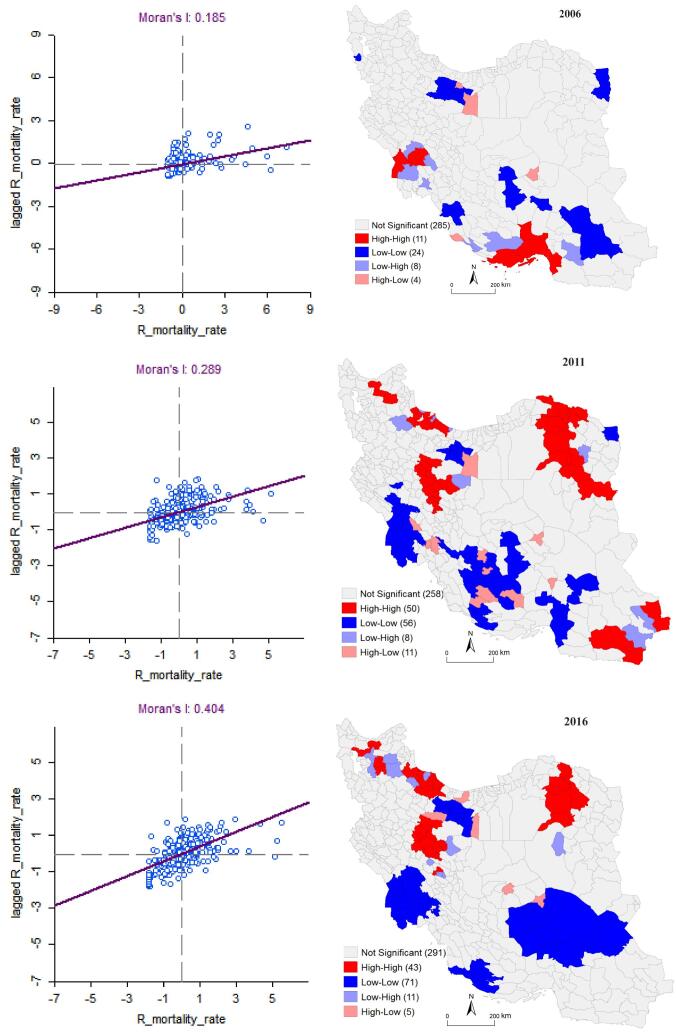
Global Moran’s I Scatterplot and LISA Maps for Rural Mortality Rate (2006, 2011, and 2016). Abbreviation: LISA, Local Indicators of Spatial Association.

 Local spatial autocorrelation provides more details of where clusters and outliers are formed. For this purpose, the local Moran’s I statistic is used, which is shown in the (Figure 5). The map on the right shows the results for the local Moran’s statistic. It is also known as LISA map or cluster and outlier analysis. LISA map shows 5 patterns based on each place and its neighborhood unit. High-high and low-low are the dominant patterns. Also, these two patterns have formed spatial clusters. The counties in red color belong to the high–high (hot spot) clusters. These are the counties with high mortality rate with similar neighbors. The counties marked in blue belong to low–low (cold spot) clusters. The diagram on the left is the Moran scatterplot. In the upper-right quadrant are cases where both the value and local average value of the attribute are higher than the overall average value. Similarly, in the lower-left quadrant are cases where both the value and local average value of the attribute are lower than the overall average value. These cases confirm positive spatial autocorrelation. Cases in the other two quadrants indicate negative spatial autocorrelation. Table shows the results related to the local Moran index.

**Table:** Local Moran’s I Cluster for Rural Mortality (2006, 2011, and 2016)

**Year**		**H-H**	**H-L**	**NS**	**LH**	**LL**	**Total**
2006	Count	11	5	286	7	23	332
	Percent	3.3	1.5	86.1	2.1	6.9	100
2011	Count	49	11	262	7	54	383
	Percent	12.8	2.9	68.4	1.8	14.1	100
2016	Count	44	6	288	10	73	421
	Percent	10.5	1.4	68.4	2.4	17.3	100

 In 2006, the high-high pattern in terms of spatial distribution is widely concentrated in the south and southwest. Eleven counties in the south include of Bandarabbas, Hajiabad, Bastak, Khamir, Minab, Qeshm, Shushtar, Aligudarz, and Hoveyzeh are in the high-high mortality rate pattern.

 In this pattern, the spatial autocorrelation is positive and there are clusters. Lower-lower counties are mainly distributed across the country. Some of the counties in the south east (Iranshahr, Kerman, Bam, Shahrebabak, Torbat-e jam, Oshnaviyeh, Zarandiyeh, Bardsir, Mehriz, Kazerun, Borazjan, and Bushehr), in the center (Mamuniyeh, Rey, Varamin, and Damavand) and finally Sarakhs in the north east are in the low-low cluster. The counties of Garmsar, Kuhbanan, Dayyer, and Shemiranat are located in the high-low pattern. These four counties with high mortality rates are adjacent to counties with low mortality rates. Spatial autocorrelation is negative and outlier. The counties in the south and south west (Qalehganj, Larestan, Asaluye, Jam, Omidiyeh, Ahvaz, Shadegan, and Dezful) are in the low-high pattern contract. These counties, which have a low mortality rate, are located in the vicinity of the cluster of high death rates. Spatial autocorrelation is negative and outlier.

 In 2011, except in Sistan and Baluchistan province in the south east country, the high-high pattern is concentrated in the northern half of the country. The high mortality rate in these regions, caused them to form a spatial cluster and have a high-high mortality pattern.

 In 2011, the low-low pattern has a trend completely opposite to the high-high pattern, so that except for the counties of Tehran province and Sarakhs county in the northeast, the clusters are formed in the southern half of the country.

 The counties of Nowshahr, Savojbolagh, Rey, Damavand, Karaj, Nazarabad, Varamin, Ahvaz, Shahrebabak, Bafq, Shiraz, Rudbar-e Jonub, Andimeshk, and Sarakhs are included in this cluster. The counties of Langerud, Bandar-e Anzali, Sib va Suran, Sarbaz, and Zanjan is located in the low-high pattern. These counties with a low death rate are located in the neighborhood of counties with a high mortality rate, so they are spatial outliers and Its spatial autocorrelation is negative. Khalkhal counties is located in the high-low pattern. The counties of Qir va Karzin, Jahrom, Masjedsoleyman, Garmsar, Rabor, Kuhbanan, Darab, Khorrambid, and Arsanjan follow the high-low pattern and are considered spatial outliers. The rural mortality rate in the rest of the counties is not significant in relation to its neighboring units.

 In 2016, the clusters have become aggregational. The high-high pattern was completely located in the northern half of the country. One of these clusters has covered all the central counties. Other counties that located into this pattern are: Golpayegan, Shirvan, Faruj, Esfarayen, Sabzevar, Bardaskan, Neyshabur, Qazvin, Rudsar, Astaneh-ye Ashrafiyeh, Khalkhal, Shabestar, Bostanabad, Hashtrud, and Varzeqan.

 The high-high pattern that was located in the south of the country in 2006 was replaced by the low-low pattern in 2016. The counties of Khuzestan province and some counties of Bushehr, south of Fars province, north of Kerman and Sistan & Baluchistan are in this pattern. Mamuniyeh, Aradan, Kuhbanan, Yazd, and Nur are also with high mortality rates in the neighborhood of counties with low mortality rates. Finally, 11 counties of Bueen Miyandasht, Ben, Boshruyeh, Kashan, Miyaneh, Sarab, Maragheh, Tabriz, Rasht, and Abhar have the pattern of low-high rural mortality rate.

 Table shows the features of the different types obtained from LISA statistics by each type of spatial clustering. The L-L class has the largest number of counties compared to other classes in the three studied periods, it has increased from 23 (6.9%) in 2006 to 73 (17.3%) in 2016. This indicates that low-mortality clusters are more prevalent in rural areas. However, the H-H class, which contains 11, 49, and 44 counties for the years 2006, 2011, and 2016, respectively, decreased in 2016.

## Discussion

 Unequal mortality is seen among different communities and geographical areas. Accurate information on the causes of death, trends and changes in rural areas is not only an important part of spatial demographic analysis, but also one of the necessities of planning, management and evaluation of the public health sector in all countries. Several studies have shown sub-national mortality differences by age such as infant mortality, or All-cause mortality such as esophageal cancer mortality, prostate cancer mortality.^45-47^ Due to the differences in the topic, the scale of the study and the methodology, it is difficult to compare. At the national level, Xiao et al findings show that from 1982 to 1990, the crude mortality rate in the country and the countryside declined, while the crude mortality rate in cities and towns rose. Then the crude mortality rate in the countryside steadily increased (1990: 6.42; 2000: 6.87; 2010: 7.46 per 1000 population).^48^ Also finding of Ahmad Kiadaliri et al have shown there were substantial differences in crude mortality rate across the provinces of Iran.^49^ A review of previous studies showed that (1) most of the researches in Iran are related to the estimation of different mortality indicators and the spatial facets has not been taken into account in the exploration and identification of spatial patterns of mortality and (2) no studies have been conducted specifically in rural areas. The purpose of this study is to identify and explore variations in the spatial patterns of rural mortality in 2016-2006. According to population statistics provided by SCI and NOCR, 151 130, 109 628, and 94 788 people died in rural areas in 2006, 2011, and 2016, respectively. During these years, the risk of rural mortality in 102, 197, and 216 counties of Iran was higher than the country excess rate. In 2006, most of these counties were concentrated in the south of the country, and in 2016, counties with a higher risk of death than the country excess rate are mostly found in the northern half of the country. After the statistical analysis of mortality, variation in spatial patterns of rural mortality have been explored and analyzed. First, the results of the spatial autocorrelation analysis obtained from the global Moran index were examined. The results of Moran’s index are positive for all three years, which shows positive spatial autocorrelation. Also, the values of Moran’s index have increased in 2016 and indicate the strengthening of the cluster spatial pattern of rural mortality rate.

 Then local spatial autocorrelation and identification of clusters and outliers were investigated. For this purpose, the local Moran index was used and the LISA map of rural mortality rate was prepared during the years 2006 to 2016. The LISA map shows the clustering of rural deaths during these years. Also, high clusters in southern counties have turned into clusters with low values, and in the northern half of the country, we see the formation of clusters with high values.

 There are limitations in our study, which are: (1) The crude mortality rate is influenced by the distribution of age. This research used basic indicators without considering the impact of gender and age differences on these indicators; (2) The data were collected from census records, which may be incomplete and contain errors, potentially leading to biased results. Issues such as undercounting, inaccurate reporting, and delayed recording are common problems with census and mortality data in Iran; (3) The changes in the number of counties during the investigated periods, which causes serious limitations for the use of spatial-temporal clustering; and (4) The results of statistical tests are strongly influenced by the shape and size of the spatial units, which can affect the results of the research. Despite this limitation, the findings of our study showed that the spatial patterns of mortality rates in rural areas have changed spatially and temporally, which can be important and significantly in terms of statistics and public health. Identifying specific clusters at high mortality risk is an attractive visual approach that helps to understand spatial processes and relationships in a region. Adopting a geographic approach in exploring spatial heterogeneity and mortality patterns is an inevitable necessity in determining public health priorities and interventions.

## Conclusion

 Mortality is one of the processes of population dynamics. Over the past several decades, as the public health of rural areas has improved and life expectancy has increased, mortality has decreased greatly. However, despite the general and national trends, there are significant differences among local levels. However, understanding the factors behind this heterogeneity requires additional studies using methodologies that scrutinize risk factors at both individual and contextual levels. Understanding the variation of rural mortality patterns over space and time at local level allows policy-makers to allocate resources more effectively. Therefore, identified clusters as high-high class require targeted interventions and increased resource allocation. The findings show that mortality rates in rural areas have dynamic spatial patterns that change over time. High-high mortality patterns have diminished in the southern half of the country while emerging in the north half of the country. These changes offer insights into the evolving healthcare needs of rural areas. These changes offer insights into the evolving healthcare needs of rural areas. Policy-makers can use this information to plan and enhance healthcare infrastructure, ensuring that it aligns with the evolving health demands of specific counties. The findings serve as a basis for evaluating the effectiveness of existing health policies during the study period. What has been done in this research provides a suitable and accurate scientific support for further fundamental research in the field of population and public health. Policy-makers can assess the impact of past interventions, identify successful strategies, and make informed adjustments to enhance the overall effectiveness of public health policies. In summary, exploring county-level mortality pattern variations in rural areas of Iran offers policy-makers a nuanced understanding of health dynamics, enabling them to make informed decisions, allocate resources efficiently, and design targeted interventions for improved public health outcomes.

## Ethical issues

 The county-level data used in this study is open-source, and the secondary data was published by the Statistical Centre of Iran and the National Organization for Civil Registration. While the data does not contain personal information, ethical considerations such as ensuring transparency in data use and methodology and respecting the representation of populations have been carefully observed to uphold the integrity of the research.

## Competing interests

 Authors declare that they have no competing interests.
